# Characteristics of Adult Outpatients and Inpatients with COVID-19 — 11 Academic Medical Centers, United States, March–May 2020

**DOI:** 10.15585/mmwr.mm6926e3

**Published:** 2020-07-03

**Authors:** Mark W. Tenforde, Erica Billig Rose, Christopher J. Lindsell, Nathan I. Shapiro, D. Clark Files, Kevin W. Gibbs, Matthew E. Prekker, Jay S. Steingrub, Howard A. Smithline, Michelle N. Gong, Michael S. Aboodi, Matthew C. Exline, Daniel J. Henning, Jennifer G. Wilson, Akram Khan, Nida Qadir, William B. Stubblefield, Manish M. Patel, Wesley H. Self, Leora R. Feldstein, CDC COVID-19 Response Team, CDC COVID-19 Response Team, Ahmed M. Kassem, Courtney N. Sciarratta, Nicole Dzuris, Paula L. Marcet, Akshita Siddula, Eric P. Griggs, Emily R. Smith, Constance E. Ogokeh, Michael Wu, Sara S. Kim

**Affiliations:** ^1^CDC COVID-19 Response Team; ^2^Vanderbilt University Medical Center, Nashville, Tennessee; ^3^Beth Israel Deaconess Medical Center, Boston, Massachusetts; ^4^Wake Forest University Baptist Medical Center, Winston-Salem, North Carolina; ^5^Hennepin County Medical Center, Minneapolis, Minnesota; ^6^Baystate Medical Center, Springfield, Massachusetts; ^7^Montefiore Medical Center and Albert Einstein College of Medicine, Bronx, New York; ^8^Ohio State University Wexner Medical Center, Columbus, Ohio; ^9^University of Washington Medical Center, Seattle, Washington; ^10^Stanford University Medical Center, Palo Alto, California; ^11^Oregon Health & Sciences University, Portland, Oregon; ^12^UCLA Medical Center, Los Angeles, California.; CDC; Public Health Institute/CDC Global Health Fellowship; CDC; CDC; CDC; Oak Ridge Institute for Science and Education; Oak Ridge Institute for Science and Education; Oak Ridge Institute for Science and Education; Oak Ridge Institute for Science and Education; Oak Ridge Institute for Science and Education.

Descriptions of coronavirus disease 2019 (COVID-19) in the United States have focused primarily on hospitalized patients. Reports documenting exposures to SARS-CoV-2, the virus that causes COVID-19, have generally been described within congregate settings, such as meat and poultry processing plants (*1*) and long-term care facilities (*2*). Understanding individual behaviors and demographic characteristics of patients with COVID-19 and risks for severe illness requiring hospitalization can inform efforts to reduce transmission. During April 15–May 24, 2020, telephone interviews were conducted with a random sample of adults aged ≥18 years who had positive reverse transcription–polymerase chain reaction (RT-PCR) test results for SARS-CoV-2 in outpatient and inpatient settings at 11 U.S. academic medical centers in nine states. Respondents were contacted 14–21 days after SARS-CoV-2 testing and asked about their demographic characteristics, underlying chronic conditions, symptoms experienced on the date of testing, and potential exposures to SARS-CoV-2 during the 2 weeks before illness onset (or the date of testing among those who did not report symptoms at the time of testing). Among 350 interviewed patients (271 [77%] outpatients and 79 [23%] inpatients), inpatients were older, more likely to be Hispanic and to report dyspnea than outpatients. Fewer inpatients (39%, 20 of 51) reported a return to baseline level of health at 14–21 days than did outpatients (64%, 150 of 233) (p = 0.001). Overall, approximately one half (46%) of patients reported known close contact with someone with COVID-19 during the preceding 2 weeks. This was most commonly a family member (45%) or a work colleague (34%). Approximately two thirds (64%, 212 of 333) of participants were employed; only 35 of 209 (17%) were able to telework. These findings highlight the need for screening, case investigation, contact tracing, and isolation of infected persons to control transmission of SARS-CoV-2 infection during periods of community transmission. The need for enhanced measures to ensure workplace safety, including ensuring social distancing and more widespread use of cloth face coverings, are warranted (*3*).

The Influenza Vaccine Effectiveness in the Critically Ill (IVY) Network is a collaboration of U.S. medical centers conducting research on vaccine effectiveness for and epidemiologic studies of influenza, and recently started conducting epidemiologic studies on COVID-19. To explore the spectrum of illness across health care settings and potential community SARS-CoV-2 exposures after issuance of national social distancing guidelines on March 16, 2020 (*4*), 11 academic medical centers in nine states conducted telephone-based surveys of a sample of patients with positive SARS-COV-2 test results during April 15–May 24, 2020 (testing dates = March 31–May 10, 2020). Medical centers submitted lists of persons with SARS-CoV-2 infection to Vanderbilt University and identified location of testing (intensive care unit [ICU], non-ICU hospital admission, emergency department [ED] without admission during the encounter, and other outpatient settings). To achieve a broadly representative cohort, selection of patients was made using site-specific stratified random sampling by location of testing. The median proportions sampled were 67% of inpatients and 53% of outpatients. Personnel from CDC telephoned patients during intervals of 14–21 days (97%) or 28–35 days (3%) after testing; up to seven call attempts were made per patient for each period. Interviews were conducted in English, Spanish, French, Creole, Portuguese, Arabic, Burmese, and Somali. Respondents or their proxies were asked to provide patient demographic and socioeconomic information, clinical signs and symptoms on the date of testing, underlying chronic conditions, and potential exposures to SARS-CoV-2 during the 2 weeks preceding illness onset (or 2 weeks before test date in patients who did not report symptoms). This 14-day exposure period was selected to encompass the estimated COVID-19 incubation period for most persons (*5*). Patients who responded at 28–35 days were asked the same questions, with the exception of signs or symptoms at the time of testing because the delay between symptom onset and interview date increased the potential for introducing recall bias.

To compare responses among patients who received inpatient and outpatient testing, descriptive statistics were analyzed, using Wilcoxon rank-sum testing for continuous variables and chi-squared or Fisher’s exact test for categorical variables. Patients with proxy respondents or who had died were excluded because details about symptoms, medical conditions, and exposure histories were frequently unknown. Statistical analyses were conducted using Stata software (version 16; StataCorp).

At least one telephone call was attempted for 798 randomly selected patients 309 inpatients [98 ICU and 211 non-ICU] and 489 outpatients [144 ED and 345 non-ED]) across the 11 sites. Among these, 544 (68%) answered calls, and 398 (50%) completed interviews. Sixty-seven (8%) patients or proxies refused, 37 (5%) were unable to complete the interview because of a language barrier, 42 (5%) requested a callback but could not be reached on further call attempts; 20 (3%) were reported to have died within 21 days of testing (nine proxy respondents interviewed and 11 refused). A total of 48 proxy interviews were excluded, leaving 350 of 398 for analysis.*

Among the 350 respondents with completed interviews, 271 (77%) were tested as outpatients (70 ED and 201 non-ED) and 79 (23%) as inpatients (17 ICU and 62 non-ICU) (Table 1). The median number of patient respondents by site was 20 (interquartile range = 11–46). The median respondent age was 43 years; 185 (53%) were female, 116 (33%) white, 73 (21%) non-Hispanic black (black), 43 (12%) non-Hispanic of another race, and 116 (33%) Hispanic. Nineteen patients reported another positive SARS-CoV-2 test result before the test date applicable to this study. Among outpatients, 8% (22 of 271) were later admitted to the hospital after having outpatient testing.

**TABLE 1:** Self-reported demographic and baseline clinical characteristics of outpatients (N = 271) and inpatients (N = 79) with SARS-CoV-2 RT-PCR–positive test results at 14–21 days or 28–35 days after testing — academic medical centers,* United States, March–May 2020

Characteristic	No. (%)			
	Total (350)	Outpatients (271)	Inpatients (79)	P-value
**Median age, yrs, (IQR)**	**43 (32–57)**	42 (31–54)	54 (36–68)	<0.001
**Female sex**	**185 (53)**	144 (53)	41 (52)	0.85
**Race/Ethnicity^†^**				0.008
White, non-Hispanic	**116 (33)**	101 (37)	15 (19)	
Black, non-Hispanic	**73 (21)**	51 (19)	22 (28)	
Hispanic	**116 (33)**	82 (30)	34 (43)	
Other, non-Hispanic	**43 (12)**	35 (13)	8 (10)	
Unknown	**2 (1)**	2 (1)	0 (0)	
**Medical Insurance**				0.85
Yes	**289 (83)**	222 (82)	67 (85)	
No	**45 (13)**	34 (13)	11 (14)	
Unknown	**16 (5)**	15 (6)	1 (1)	
**Education level**				0.83
Less than college	**177 (51)**	135 (50)	42 (53)	
Some college or more	**154 (44)**	119 (44)	35 (44)	
Unknown	**19 (5)**	17 (6)	2 (3)	
**Annual household income**				0.003
<$25,000	**56 (16)**	34 (13)	22 (28)	
$25,000–$49,000	**92 (26)**	77 (28)	15 (19)	
$50,000–$74,000	**33 (9)**	27 (10)	6 (8)	
>$74,000	**57 (16)**	49 (18)	8 (10)	
Unknown/Refused to answer	**112 (32)**	84 (31)	28 (35)	
**Underlying medical condition (334)^§^**				
Number, median (IQR)	**1 (0–2)**	1 (0–2)	2 (1–3)	<0.001
Any cardiac disease	**100 (30)**	69 (27)	31 (41)	0.019
Hypertension	**97 (29)**	67 (26)	30 (39)	0.023
Coronary artery disease	**10 (3)**	5 (2)	5 (7)	0.037
Congestive heart failure	**9 (3)**	3 (1)	6 (8)	0.005
Any respiratory disease	**65 (20)**	40 (16)	25 (33)	0.001
Asthma	**55 (16)**	36 (14)	19 (25)	0.022
COPD	**18 (5)**	6 (2)	12 (16)	<0.001
Diabetes	**51 (15)**	28 (11)	23 (30)	<0.001
Obesity (BMI ≥30 kg/m2)	**67 (20)**	47 (18)	20 (26)	0.13
Chronic kidney disease	**14 (4)**	8 (3)	6 (8)	0.067
Chronic liver disease	**11 (3)**	5 (2)	6 (8)	0.011
Immunosuppressive condition	**22 (7)**	16 (6)	6 (8)	0.60
Rheumatologic/Autoimmune condition	**28 (8)**	20 (8)	8 (11)	0.45
Neurologic condition	**16 (5)**	9 (4)	7 (9)	0.041
Blood disorder	**12 (4)**	7 (3)	5 (7)	0.11
Psychiatric disorder	**69 (21)**	52 (20)	17 (23)	0.65
**Ever used tobacco^¶^**	**104 (31)**	77 (30)	27 (36)	0.36
Current tobacco use (among ever users)	**17 (17)**	15 (20)	2 (7)	0.23
Current alcohol use**	**112 (34)**	89 (35)	23 (30)	0.45

**Abbreviations**: BMI = body mass index; COPD = chronic obstructive pulmonary disease; IQR = interquartile range; RT-PCR = reverse transcription–polymerase chain reaction.

* Patients were sampled from 11 academic medical centers in nine states (University of Washington [Washington], Oregon Health and Sciences University [Oregon], University of California Los Angeles and Stanford University [California], Hennepin County Medical Center [Minnesota], Vanderbilt University [Tennessee], The Ohio State University [Ohio], Wake Forest University [North Carolina], Montefiore Medical Center [New York], Beth Israel Deaconess Medical Center and Baystate Medical Center [Massachusetts]).

^†^ Other non-Hispanic included two persons who reported being American Indian/Alaska Native, 25 Asian, three Native Hawaiian/Other Pacific Islander, and 18 Other; five reported both Asian and Other for race. Other race group combined because of comparatively low numbers in these groups compared with other race/ethnicity groups.

^§^ Excluding 16 (5%) patients who did not answer questions about underlying medical conditions; for those who answered questions about underlying conditions, some respondents were missing data on congestive heart failure (one), obesity (three), rheumatologic/autoimmune conditions (one), neurologic conditions (one), and psychiatric conditions (two); denominators used to calculate proportions of respondents with individual underlying medical conditions excluded patients who have missing data for the condition.

**^¶^** Unknown for 17 (14 outpatients and three inpatients); among those who had ever used tobacco products, one did not state whether they were a current tobacco user.

** Unknown for 19 (16 outpatients and three inpatients).

## Demographic and Baseline Health Characteristics

Compared with outpatients, inpatients were older (median age = 54 versus 42 years; p<0.001) and differed by race/ethnicity (p = 0.008) and annual household income (p = 0.003). Inpatients were less likely to be white (19% versus 37%) and more likely to have annual household income <$25,000 (28% versus 13%). Inpatients also had more underlying chronic conditions (median = two) than did outpatients (median = one) (p<0.001), including cardiovascular conditions, chronic respiratory disease, and diabetes.

## Reported Symptoms

Among 316 (90%) respondents who answered questions on symptoms and did not report a previous positive SARS-CoV-2 test result,^†^ 292 (92%) reported one or more symptoms on the date of SARS-CoV-2 testing (Table 2), including 238 (96%) of 248 outpatients and 54 (79%) of 68 inpatients. Both inpatients and outpatients reported a similar number of symptoms, but inpatients were more likely to describe dyspnea (72% versus 32%; p<0.001) and less likely to report loss of smell or taste (43% versus 59%; p = 0.030). Fewer symptomatic inpatients (39%, 20 of 51) reported a return to baseline level of health at 14–21 days than did symptomatic outpatients (64%, 150 of 233) (p = 0.001).

**TABLE 2:** Symptoms reported on the date of SARS-CoV-2 test in outpatients and inpatients who tested positive for SARS-CoV-2 (N = 316) at 14–21 days or 28–35 days after testing — 11 academic medical centers,* United States, March–May 2020

Characteristic^†^	No. (%)			P-value
	All (316)	Outpatients (248)	Inpatients (68)	
**Reported any symptom^§^**	**292 (92%)**	238 (96%)	54 (79%)	N/A
**Symptoms reported^¶^**				
Median no. of symptoms (IQR)	**7 (4–10)**	7 (4–10)	8 (4–10)	0.18
Fever	**167 (57)**	131 (55)	36 (68)	0.086
Shortness of breath	**114 (39)**	76 (32)	38 (72)	<0.001
Cough	**182 (63)**	147 (62)	35 (69)	0.36
Productive	**91 (50)**	72 (49)	19 (54)	0.57
Bloody	**16 (9)**	10 (7)	6 (17)	0.054
Chest pain	**82 (28)**	60 (25)	22 (42)	0.014
Pleuritic pain	**61 (76)**	43 (74)	18 (82)	0.47
Abdominal pain	**55 (19)**	42 (18)	13 (25)	0.20
Nausea	**93 (32)**	73 (31)	20 (38)	0.28
Vomiting	**35 (12)**	24 (10)	11 (21)	0.027
Diarrhea	**109 (38)**	91 (38)	18 (35)	0.61
Chills	**156 (54)**	124 (52)	32 (60)	0.29
Body aches	**167 (58)**	138 (58)	29 (56)	0.72
Headache	**171 (60)**	146 (62)	25 (48)	0.062
Confusion	**41 (14)**	35 (15)	6 (12)	0.53
Fatigue	**198 (69)**	164 (70)	34 (65)	0.54
Congestion	**110 (38)**	91 (39)	19 (37)	0.77
Sore throat	**89 (31)**	73 (31)	16 (31)	0.97
Loss of smell	**140 (49)**	122 (52)	18 (35)	0.031
Loss of taste	**143 (50)**	122 (52)	21 (41)	0.16
Loss of smell, taste, or both	**163 (56)**	140 (59)	23 (43)	0.030
Returned to baseline health by interview date**	**170 (60)**	150 (64)	20 (39)	0.001

**Abbreviations**: IQR = interquartile range; N/A = not applicable.

* Patients were sampled from 11 academic medical centers in nine states (University of Washington [Washington], Oregon Health and Sciences University [Oregon], University of California Los Angeles and Stanford University [California], Hennepin County Medical Center [Minnesota], Vanderbilt University [Tennessee], The Ohio State University [Ohio], Wake Forest University [North Carolina], Montefiore Medical Center [New York], Beth Israel Deaconess Medical Center and Baystate Medical Center [Massachusetts]).

^†^ Among 350 patients who had positive test results for SARS-CoV-2 and responded, 19 (5%) who reported a previous positive SARS-CoV-2 test result before the current test (10 outpatients and nine inpatients) were excluded. An additional 15 (4%) were excluded who did not answer symptom questions during the call 14–21 days after testing (five) or who only responded to the follow-up call at 28-35 days after testing, which did not include symptom questions (10).

^§^ Four percent (10 of 250) of outpatients reporting no symptoms were tested because of a job requirement (four), being a close contact of a COVID-19 patient (three), requirement before a scheduled surgery (two), and voluntarily tested because of advanced age and underlying medical conditions (one); 21% (14 of 66) of inpatients reporting no symptoms were tested while hospitalized for unrelated reasons, including six pregnant women hospitalized for delivery and eight for other reasons.

^¶^ Among 292 respondents who reported one or more symptoms, some respondents were missing data on individual symptoms: fever (one), shortness of breath (one), cough (three), chest pain (three), abdominal pain (four), nausea (three), vomiting (three), diarrhea (three), chills (two), body aches (four), headache (five), confusion (six), fatigue (five), congestion (five), sore throat (five), loss of smell (six), loss of taste (seven); denominators used to calculate proportions of respondents with individual symptoms excluded patients who had missing data for the symptom.

** Eight responses on return to baseline health were missing.

## Exposures

Among 339 (97%) participants who provided exposure histories, 46% (153 of 332) reported a close case contact, defined as being within 6 feet of someone with a diagnosis of COVID-19, during the 2 weeks preceding illness onset or the date of testing for asymptomatic patients (Table 3). This was most commonly a family member (45%, 69 of 153) or a work colleague (34%, 52 of 153). Seven of the 339 participants were missing data in their case contact histories.

**TABLE 3:** Exposures and behaviors in the 2 weeks preceding illness onset in outpatients and inpatients who had positive test results for SARS-CoV-2 (N = 339) at 14–21 days or 28–35 days after testing — 11 academic medical centers,* United States, March–May 2020^†^

Characteristic^§^	No. (%)			
	All (339)	Outpatients (262)	Inpatients (77)	P-value
**Contact (≤6 feet) with COVID-19 patient [might have multiple]**	**153 (46)**	129 (50)	24 (32)	0.004
**Contact**				
Family member	**69 (45)**	56 (43)	13 (54)	
Work colleague	**52 (34)**	47 (36)	5 (21)	
Friend	**15 (10)**	14 (11)	1 (4)	
Other**^¶^**	**29 (19)**	22 (17)	7 (29)	
**Type of residence**				<0.001
Single family home	**211 (62)**	176 (67)	35 (45)	
Apartment	**94 (28)**	66 (25)	28 (36)	
Long-term care facility	**4 (1)**	0 (0)	4 (5)	
Group home	**1 (<1)**	0 (0)	1 (1)	
Other	**29 (9)**	20 (8)	9 (12)	
**Lives with others**	**303 (89)**	232 (89)	71 (92)	0.36
No. of other household members, median (IQR)	**3 (1–4)**	3 (1.5–4)	2 (1–4)	0.49
**Employed**	**212 (64)**	180 (70)	32 (42)	<0.001
**If employed, worked outside home within last 2 wks**				0.49
Every day	**118 (59)**	102 (60)	16 (52)	
2–3 times per wk	**38 (19)**	31 (18)	7 (23)	
Once per wk	**6 (3)**	6 (4)	0 (0)	
Never	**39 (19)**	31 (18)	8 (26)	
**If employed, ability to telework**	**35 (17)**	32 (18)	3 (10)	0.25
**If employed, worked in health care facility**	**53 (25)**	46 (26)	7 (23)	0.72
**Total number of daily contacts, median (IQR)**	**5 (2–10)**	5 (3–13)	3 (1–10)	0.013
**Frequency of interaction with others outside of home**				<0.001
Every day	**130 (41)**	113 (47)	17 (23)	
2–3 times per wk	**65 (21)**	47 (19)	18 (24)	
Once per wk	**38 (12)**	32 (13)	6 (8)	
Never	**83 (26)**	50 (21)	33 (45)	
**Days going out for groceries**				0.071
Every day	**7 (2)**	4 (2)	3 (4)	
2–3 times per wk	**85 (27)**	65 (27)	20 (27)	
Once per wk	**120 (38)**	100 (41)	20 (27)	
Never	**107 (34)**	75 (31)	32 (43)	
**Attended gathering with >10 persons**	**28 (8)**	21 (8)	7 (9)	0.77
**Used public transportation**	**23 (7)**	12 (5)	11 (15)	0.003

**Abbreviations**: COVID-19 = coronavirus disease 2019; IQR = interquartile range.

* Patients were sampled from 11 academic medical centers in nine states (University of Washington [Washington], Oregon Health and Sciences University [Oregon], University of California Los Angeles and Stanford University [California], Hennepin County Medical Center [Minnesota], Vanderbilt University [Tennessee], The Ohio State University [Ohio], Wake Forest University [North Carolina], Montefiore Medical Center [New York], Beth Israel Deaconess Medical Center and Baystate Medical Center [Massachusetts]).

^†^ Exposures were elicited in 2 weeks preceding illness onset or 2 weeks preceding testing for asymptomatic patients.

^§^ Of 350 patient respondents, 339 were included; 11 (3%) were excluded for not answering any of the exposure-related questions; for individual exposures in 339 included respondents, some respondents were missing data on close contact with a person with a COVID-19 case (seven), being employed (six), working outside the home (11), ability to telework (three), working at a health care facility (one), average number of daily contacts outside the home (15), frequency of interaction with others outside the home (23), days going out for groceries (20), attendance at gathering with ≥10 persons (six), and use of public transportation (six); denominators used to calculate proportions of respondents with individual exposures or behaviors exclude patients with missing data for the exposure or behavior.

^¶^ Other included exposures within health care settings (18), assisted living facilities (six), neighbors (two), clients at work (one), exposure at a correctional facility (one), and roommate at long-term care facility (one); among 24 exposures in health care settings or assisted living facilities, 22 were reported among persons who worked in a health care facility.

Approximately two thirds (64%, 212 of 333) of participants were employed; however, only 35 of 209 (17%) were able to telework. Outpatients were more likely to report being employed than were inpatients (70% versus 42%; p<0.001) and interacted with persons outside the home more frequently (p<0.001). Among employed participants, 53 (25%) reported working in health care.

## Discussion

Few studies have systematically collected data on COVID-19 patients from varied health care settings in the United States. In this multistate telephone-based survey of 350 U.S. COVID-19 outpatients and inpatients, inpatients were typically older and had more underlying chronic conditions, findings that have been previously observed with both COVID-19 and influenza patients (*6*–*8*). Compared with outpatients, inpatients reported lower household incomes and were less likely to be white. Differences by race/ethnicity are consistent with those reported previously (*9*) (e.g., 43% of inpatients were Hispanic, and 28% were black), although in this descriptive analysis no adjustment for other factors was made to evaluate any independent association between race/ethnicity and COVID-19 severity.

Approximately one third of symptomatic outpatients reported that they had not returned to baseline health by the interview date 14–21 days after testing positive for SARS-CoV-2 infection. In comparison, almost all outpatient working adults with laboratory-confirmed influenza reported returning to normal activities within 14 days of illness onset during the 2012–13 influenza season (*10*).

Fewer than one half of patients were aware of recent close contact with someone with COVID-19, highlighting a need for increased screening, case investigation, contact tracing, and isolation of infected persons during periods of community transmission. This finding suggests that ensuring social distancing and more widespread use of cloth face coverings are warranted (*3*). A majority of COVID-19 patients reported working during the 2 weeks preceding illness, and few had the ability to telework, underscoring the need for enhanced measures to ensure workplace safety.

The findings in this report are subject to at least six limitations. First, given that the survey was telephone-based, some nonresponse bias is possible. Patients with more severe illnesses might have still been hospitalized at the time of the survey or might have died, resulting in a higher proportion of nonrespondents among patients with more severe illness. Estimates of the frequency of clinical characteristics should therefore be interpreted with caution. Second, patients were sampled from academic medical centers with differing numbers of respondents; therefore, patients in this study are not representative of cases nationwide. With limited testing capacity, some groups (e.g., health care and other essential workers) might also have been preferentially tested. Third, data were obtained by self-report and might be subject to recall bias. Fourth, this survey documented a cross-section of symptoms reported on the date of testing, and symptoms might have changed during the course of illness. In addition, a few patients reported an earlier positive test result, which might have led to misclassification of test setting; however, this was infrequent (5%). Fifth, no adjustment for other factors to determine whether variables were independently associated with illness severity was made. Finally, a small proportion of respondents were asymptomatic at the time of testing. However, comparisons including demographics and exposure histories were similar when the analysis was restricted to only patients who reported symptoms.

This study provides insights into epidemiologic characteristics of patients with laboratory-confirmed COVID-19 during March–May 2020, documenting differences between patients with medically attended outpatient and inpatient illness regarding demographic characteristics, baseline underlying chronic conditions, symptoms, and exposures that could be used to target public health interventions. In addition, among symptomatic respondents, inpatients and outpatients with COVID-19 reported similar numbers of symptoms, but different types of symptoms as previously described.^§^ Thus, a range of symptoms should prompt testing for SARS-CoV-2. The wide range of symptoms reported, and the lack of known COVID-19 contact in 54% of patients, underscores the need for isolation of infected persons, contact tracing and testing during ongoing community transmission, and prevention measures including social distancing and use of cloth face coverings.

**Summary**

**What is already known about this topic?**

Exposures to SARS-CoV-2 have commonly been described in congregate settings rather than broader community settings.

**What is added by this report?**

In a multistate telephone survey of 350 adult inpatients and outpatients who tested positive for SARS-CoV-2 infection, only 46% reported recent contact with a COVID-19 patient. Most participants’ contacts were a family member (45%) or a work colleague (34%). Two thirds of participants were employed; only 17% were able to telework.

**What are the implications for public health practice?**

Case investigation, contact tracing, and isolation of infected persons are needed to prevent ongoing community transmission, given the frequent lack of a known contact. Enhanced measures to ensure workplace safety, including social distancing and more widespread use of cloth face coverings, are warranted.

## Footnotes

* Patients with a proxy respondent were more likely to have been hospitalized (83% versus 23%) and to be older (median age = 67 versus 43 years) than were patients who responded for themselves.

† Symptoms were asked in reference to the date of SARS-CoV-2 testing. Given uncertainty in reference date for patients with a previous positive SARS-CoV-2 RT-PCR test result, symptoms were not reported for these patients.

§ https://www.cdc.gov/coronavirus/2019-ncov/symptoms-testing/symptoms.html.
